# Integrating artificial intelligence across the cancer drug discovery pipeline using a design-test-refine workflow

**DOI:** 10.3389/fonc.2026.1937655

**Published:** 2026-09-03

**Authors:** Jinming Bai, Jessica Sarah Faure, Tawfeeq Ahmed Khalfe, Rongqin Huang, Carly Ann Burmeister, Annick van Niekerk, Sharon Prince

**Affiliations:** 1 Division of Cell Biology, Department of Human Biology, Faculty of Health Sciences, University of Cape Town, Cape Town, South Africa; 2 Department of Chemistry, Faculty of Science, University of Cape Town, Cape Town, South Africa; 3 School of Pharmaceutical Sciences, Key Laboratory of Smart Drug Delivery (Ministry of Education), National Key Laboratory of Advanced Drug Formulations for Overcoming Delivery Barriers, Fudan University, Shanghai, China

**Keywords:** AI-driven drug design, biomarker identification, cancer therapeutics, combination therapy, drug response prediction, generative molecular design, perturbational transcriptomics, target discovery

## Abstract

Artificial intelligence-driven drug design (AIDD) is increasingly transforming cancer drug discovery, but its applications are often considered as individual computational tasks rather than interconnected stages of the drug discovery pipeline. This mini-review addresses this gap by firstly identifying the following four interconnected stages of anticancer drug development (1) target identification and biomarker-guided prioritization (2), structure-based and generative molecular design (3), perturbational mechanism-of-action assessment, and (4) drug response, resistance, and combination prioritization. It, secondly, describes how AI can be integrated across these four stages in a design-test-refine workflow where AI-generated predictions are progressively evaluated and refined through experimental and patient-relevant evidence. We emphasize that the role of AI in predicting target dependency, molecular activity, mechanism-of-action, or drug response should be to guide rather than replace experimental discovery or clinical judgment. Key limitations to the application of AIDD in cancer are highlighted, including in training and benchmarking data, in accounting for biological heterogeneity, as well as in model generalization. Importantly, robust validation across increasingly complex cancer models is required and it is proposed that AIDD should be used to prioritize testable treatment predictions. Ultimately, translationally useful AIDD workflows should move beyond isolated predictions toward iterative, biologically informed therapeutic development, to form a holistic design-test-refine workflow.

## 1 Introduction

Artificial intelligence-driven drug design (AIDD) is commonly associated with virtual screening of known compounds, protein-ligand modeling, and *de novo* compound generation. However, in therapeutics, especially for cancer, using AIDD solely is insufficient and it is important to understand the molecular complexity of the specific cancer to be treated. This allows for the identification of biologically justified therapeutic targets which can improve drug efficacy and reduce off-target effects. In this regard, large-scale Clustered Regularly Interspaced Short Palindromic Repeats (CRISPR) and RNA interference (RNAi) dependency maps, multi-omics integration, and translational dependency modeling provide an important foundation for AI-assisted target identification and patient stratification. Once a target for an intended patient group is defined, structure prediction, protein-ligand modeling, and generative molecular design can propose compounds that can be synthesized and tested. This also takes into consideration the potency, selectivity, and feasibility of developing the compounds. However, predicted drug-target engagement or activity does not always equate to a therapeutic effect. Therefore, an additional layer of evidence is required to assess whether a drug inhibits the expected oncogenic target/pathway and or alters the cellular phenotype. The Connectivity Map (CMap), Library of Integrated Network-Based Cellular Signatures (LINCS) L1000, and drug-induced transcriptomic prediction models provide this by predicting the mechanism-of-action inference, signature reversal and/or screening phenotypes. Furthermore, results from pharmacogenomic, single cell, organoid, patient-derived xenograft (PDX), and clinically annotated datasets are increasingly used in combination with AI tools to learn and predict drug response, resistance, and combination therapies to produce more translationally relevant outputs.

**Figure 1:**
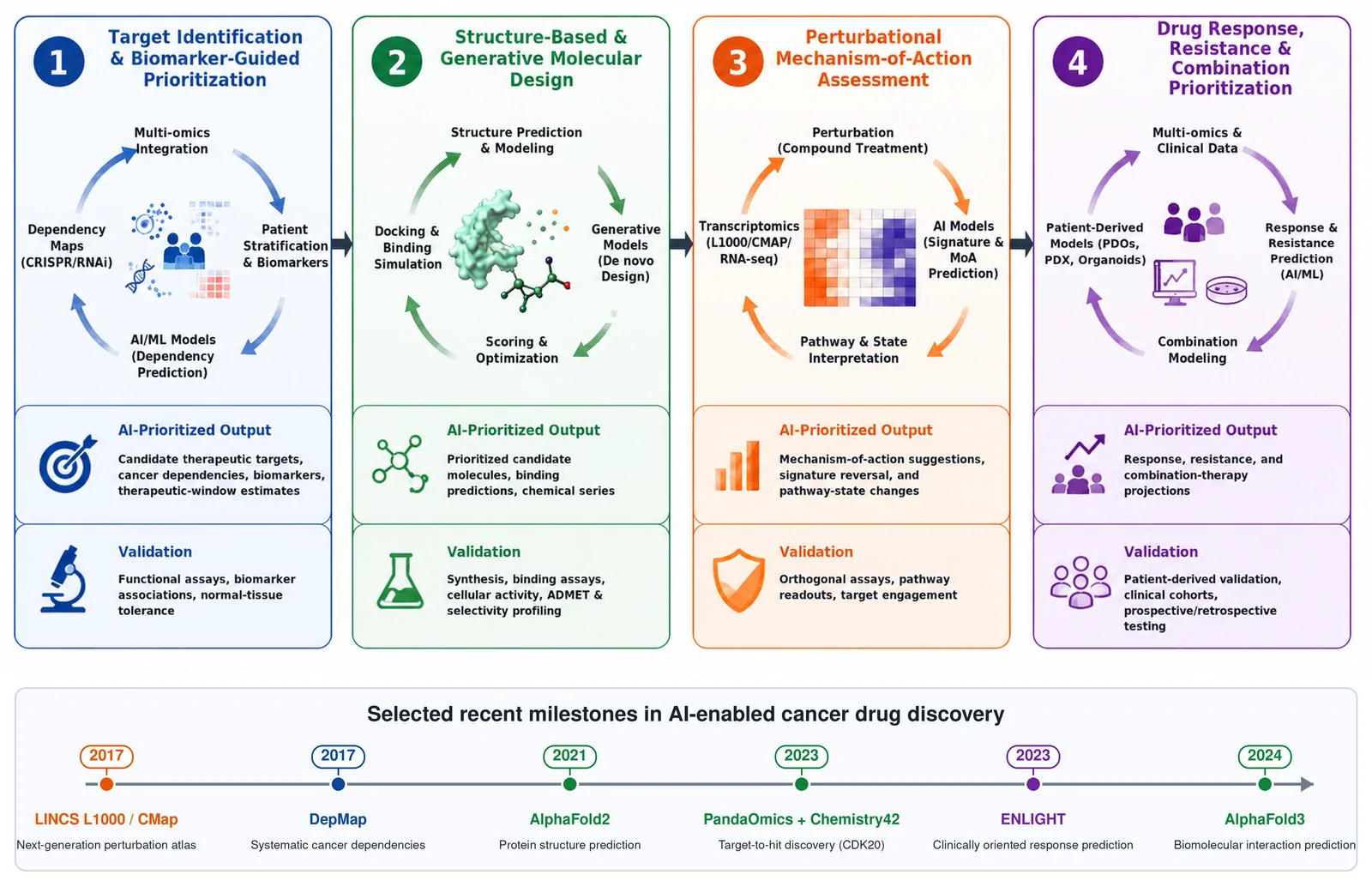
A design-test-refine workflow of AIDD in cancer therapeutics. The workflow shows the four key stages of AIDD in cancer and how they are linked. Each stage follows an iterative prediction-validation-refinement cycle, with experimental and patient-relevant evidence feeding back into target selection and molecular optimization.

There are comprehensive reviews describing the application of AI within individual stages of the cancer drug discovery pipeline (1–5). For example, Gangwal and Lavecchia have reviewed the application of AI in the generative molecular design stage (3), Szalai and Veres have reviewed the application of AI in the perturbational transcriptomics stage (4), and Partin et al, have described the application of AI in the drug response prediction stage (5). Less attention has therefore been given to how AI approaches can refine progression from one stage to the next in the drug discovery pipeline. The current mini-review addresses this gap and is the first to describe how AI approaches can be applied across the following four successive and complementary stages of anticancer drug development: target identification, molecular design, perturbation-based functional assessment, and drug response, resistance, and combination prioritization (**Figure 1**). We propose that this forms a design-test-refine workflow for AIDD in cancer therapeutics and at each stage, we consider the contribution of AI, the experimental evidence required to validate outputs, and the translational challenges that may limit clinical application (**Table 1**). By linking these stages within a single workflow, we highlight how AIDD in cancer can move beyond hypothetical and/or individual predictions, toward a more efficient and biologically relevant system.

**Table 1:** AI-prioritized outputs, validation standards, and dominant translational risks across the four cancer AIDD stages.

Stage no	AIDD stage	AI-prioritized output	Validation standard	Dominant translational risk
1	Target identification and biomarker-guided prioritization	Candidate therapeutic targets, cancer dependencies, biomarkers, and therapeutic-window estimates	Functional assays, patient-correlative analyses, biomarker measurability, and normal-tissue tolerability	Cell-line dependencies may not translate into patient efficacy
2	Structure-based and generative molecular design	Prioritized candidate molecules, binding predictions, and optimized chemical series	Synthesis, binding assays, cellular activity, ADMET, selectivity, and resistance-relevant assays	Docking bias, poor developability, and inadequate selectivity
3	Perturbation-based mechanism-of-action assessment	Mechanism-of-action suggestions, signature reversal, and pathway-state changes	Defensible generalization evaluation, experimental perturbation, and independent target-binding or pathway assays	Transcriptional similarity does not, by itself, demonstrate a mechanism
4	Drug response, resistance, and combination prioritization	Response, resistance, and combination-therapy projections	Patient-derived models, external cohorts, clinically annotated validation, and prospective testing where feasible	Benchmark overfitting; toxicity and scheduling may be inadequately modeled

## 2 Target identification and biomarker-guided prioritization

The first stage in AIDD for cancer is to identify suitable therapeutic targets for validation. This stage should consider the cancer type, molecular subtype, and biomarker-defined patient group. Dependency maps derived from large-scale CRISPR and RNAi experiments, including DepMap and Project Score, have enabled the systematic identification of genes required for cancer cell survival across molecularly annotated cell lines (6–8). This has been extended by AI and machine-learning methods which integrate genomics, proteomics, and drug-response features to infer cancer dependencies for therapeutic target validation. DeepDEP is a machine-learning model that uses multi-omics data as inputs to predict dependency profiles for tumors as outputs that have not been experimentally screened. This extends functional genomics to the patient by identifying a gene on which individual tumors might depend which facilitates the identification of patient-specific treatments (9). More recently, cancer gene dependency models trained on DepMap cell lines have been applied to patient tumors, xenograft, and healthy tissue datasets, including The Cancer Genome Atlas (TCGA), Patient-Derived Xenograft Encyclopedia (PDXE), and the Genotype-Tissue Expression (GTEx) respectively. Together, this allows for the comparison of tumor dependency with normal tissue tolerability to ascertain whether a target is both essential for the cancer phenotype and safe to inhibit (10).

Another important use for AI is to determine synthetic lethality (SL) gene pairs, in which loss of one gene alone is tolerated but simultaneous loss is lethal (11). Early AI tools such as DAISY, ISLE, and SLIdR inferred SL relationships statistically by mining tumor multi-omics, perturbation screens, and patient clinical data (11–13). To better identify SL pairs, contemporary machine learning integrates these tools with protein-protein interaction, Gene Ontology, and pathway information and contextualize them within a biological network. Examples of such machine learning include the multi-view graph auto-encoder SLMGAE, the knowledge graph neural network KG4SL, and KR4SL. Ensemble models such as ELISL provide more advanced information by incorporating protein sequence features to reduce reliance on cancer type-specific omics that are often scarce or noisy (14–17). The outputs of these models can be translated into experimentally confirmed hits. For example, 46.8% of SL gene pairs prioritized by the graph transformer model MLEC-iSL were confirmed in a CRISPR double-knockout screen (18). Systematic benchmarking of twelve SL prediction methods, however, shows that their prediction accuracy drops sharply for genes not seen during training and for context-specific SL interactions (19). Predicted SL gene pairs should, therefore, be experimentally validated using cell-culture models, organoids, xenografts, and/or patient-associated analyses.

AI models that can integrate proteomic and phosphoproteomic data can further refine prioritization of SL gene pairs. An example is the deep learning pipeline DeeProM which was built on the DeepOmicNet architecture and trained on the proteomes of 949 cancer cell lines that revealed thousands of protein biomarkers of cancer vulnerabilities that were not significant at the transcript level (20). Furthermore, tools such as INKA use data from single-sample phosphoproteomes to rank inferred kinase activities and are used to guide kinase inhibitor selection in patient-derived xenografts (21). The inferred kinase activities, however, vary appreciably between inference methods and require careful interpretation (22). Consistent with this, pan-cancer proteomic and phosphoproteomic maps have shown that protein abundance and signaling activity can predict essential genes and therapeutic response (20, 23).

In summary, AIDD streamlines target identification by prioritizing candidate targets, biomarkers, and relevant validation models. However, target selection must also consider druggability, biomarker measurability, genotype selectivity, and normal tissue tolerability. This biological foundation informs the structure-based and generative molecular design that follows.

## 3 Structure-based and generative molecular design

Once a therapeutic target has been identified, the next step is to design or discover a compound that interacts with the target to alter the disease state. Traditional synthesis and testing of compounds are costly and time-consuming, and incorporating AI into these processes has been invaluable. Firstly, AI has expanded the available target library. An example is AlphaFold2 which is a deep-learning-based model developed by DeepMind. It can accurately predict previously unresolved protein structures, thereby significantly expanding the proteome available for structure-based target discovery. DeepMind later introduced AlphaFold3 which extended this predictive power to include the structure and biomolecular interactions of proteins, nucleic acids, ligands, ions, and modified residues (24, 25). In cancer drug discovery, these advances are particularly relevant for kinases involved in oncogenic signaling and DNA repair, in which druggability depends on conformational states, allosteric pockets, or mutation-specific cavities. While accessing a wider protein library is valuable, there are still some inherent limitations in the current protein structure prediction models that cannot be ignored. Many of these challenges arise from the dynamic nature of proteins, complications from post-translational modifications, or the fact that current models cannot accurately predict the structure of membrane proteins (26, 27).

Secondly, AI-generative models have contributed to *de novo* drug development. Here, AI-assisted docking and protein-ligand modeling increasingly complement conventional physics-based methods. Unlike conventional docking against a rigid receptor, AI-assisted docking such as diffusion-based and equivariant generative approaches explore multiple plausible ligand poses, account for protein-ligand flexibility, and model conformational changes induced by ligand binding (28, 29). In addition, graph neural networks, molecular language models, reinforcement learning, and diffusion models optimize multiple drug-like properties, including potency, selectivity, synthetic accessibility, and toxicity (30, 31). Benchmark studies, however, caution that AI-assisted docking metrics do not necessarily indicate physically valid conformations or suit novel targets lacking prior drug interaction data (32, 33).

A study by Ren et al. exemplifies the use of AI platforms in stage 2 of the design-test-refine approach to the AIDD pipeline for cancer therapeutics (34). To identify a target to hit and a hit to target for hepatocellular carcinoma, the authors combined the AI platforms, PandaOmics, AlphaFold and Chemistry42. PandaOmics nominated CDK20, AlphaFold predicted its previously unresolved structure, and Chemistry42 designed candidate binders to CDK20. Through this AI-powered pipeline, a first-in-class CDK20 hit was designed, synthesized, and experimentally validated within 30 days of target selection (34). Similarly, Chemistry42 was used to prepare a novel Polθ inhibitor for BRCA-deficient cancers (35). Quantum machine learning (ML) combined with classical generative AI has also been used to design KRAS inhibitors. Here the addition of quantum effects allowed the model to explore high-dimensional probability distributions more efficiently than conventional generative models, leading to better proposed structures (36).

Finally, AI can virtually screen large compound libraries with improved success where conventional structure-based drug design has failed due to shallow pockets, mutation-induced conformational changes, or where limited structural data was available. One such example is the search for inhibitors of the epidermal growth factor receptor (EGFR) with T790M mutations, which often emerge after prolonged treatment of non-small cell lung cancer (NSCLC) with erlotinib (37). By combining five different ML algorithms, Zhou et al. screened 70,413 compounds against T790M EGFR mutants in silico and experimentally validated the lead compound *in vitro* and *in vivo* (38). More recently, generative chemistry also contributed to the discovery of a bifunctional proteolysis-targeting chimera (PROTAC), D16-M1P2, which is directed against Protein Kinase Membrane-Associated Tyrosine and Threonine (PKMYT1), a SL target for cancers where it is overexpressed (39). Indeed, treatment of breast cancer cells that overexpress PKMYT1 with D16-M1P2 resulted in antiproliferative activity in a mouse xenograft model.

Overall, AI accelerates hit discovery through structure prediction, generative molecular design, and virtual screening while reducing experimental costs by approximately 15-22% (27). However, AI-generated candidates remain susceptible to unrealistic drug conformations, excessive ligand strain, poor synthetic accessibility, weak absorption, distribution, metabolism, excretion, and toxicity (ADMET) properties, and off-target liabilities (30, 40). Moreover, AI-based drug discovery is limited by data scarcity and the sheer scale of chemical space. Current models explore only a fraction of the >10^60^ possible molecules, leading them to reproduce variations of known scaffolds rather than generate novel chemotypes with meaningful therapeutic potential (41, 42). It is therefore important that AI-generated compounds be extensively experimentally validated and that the terminology used reflects the level of validation and distinguishes computationally generated candidate compounds from experimentally validated hits, leads, and preclinical candidates (30, 40).

## 4 Perturbational transcriptomics to determine mechanism-of-action

The ability of a drug to bind its target does not, on its own, guarantee a therapeutic meaningful phenotype. It should also alter the activity of the intended biological pathway which can be confirmed experimentally by changes in gene expression profile. Perturbational transcriptomics addresses this by measuring gene expression changes following a specific intervention such as a drug treatment. CMap introduced the foundational concept that small molecules, genes, and diseases can be connected through gene expression signatures i.e. the pattern of genes that a given perturbation up- and down-regulates (43). LINCS L1000 expanded this capacity by measuring more than one million gene expression profiles induced by perturbations. This enables systematic comparison of drug- and gene-induced states across compounds, cell lines, and conditions (44). In cancer research, this framework supports two main uses. Firstly, it nominates compounds predicted to reverse a tumor-associated transcriptional state by an approach called signature reversal (45). Secondly, through mechanism-of-action inference it matches a compound’s profile against reference chemical or genetic perturbations to predict candidate drug targets (44, 46).

Recent AI models, such as DeepCE and CIGER, have moved the field from querying existing gene expression profiles toward predicting gene expression changes induced by previously untested perturbations. These models have been trained on large sets of measured perturbation profiles but differ in what they predict. Whereas DeepCE estimates the magnitude by which the expression of each gene changes, CIGER predicts the rank order of genes from most up- to most down-regulated (47, 48). Later models have placed greater emphasis on cellular context, recognizing that the same compound can induce different responses across various cell states. Indeed, MultiDCP and related models use baseline gene expression from untreated cell lines to generate context-specific predictions (49). In addition, TranSiGen and PRnet combine compound structure with baseline gene expression to model perturbation responses. Their predicted profiles then support phenotype-driven screening, drug repurposing, and candidate validation in cancer models (50, 51). Most recently, XPert introduced a biologically informed dual-branch transformer that separately models pre- and post-perturbation states, as well as dose-time dynamics. This benchmarks prior models against simple baselines (52).

The above models, however, remain constrained by the quality and biological context of their training data. L1000, for example, directly measures 978 landmark genes and computationally infers the expression of the remaining genes (44). Experimental factors such as plate and batch effects can reduce measurement consistency, while biological variability, including differences in dose, cellular state, and cancer type can alter the observed drug-induced transcriptional responses and limit reproducibility across replicates. Consequently, signatures generated in established cancer cell lines may not fully capture responses in all cancer types or patient tumors, particularly where differences in cellular composition and tumor microenvironment influence drug response (44, 49–52). Emerging spatial perturbation methods may help address some of these limitations by preserving tissue architecture and distinguishing cell-autonomous effects from perturbation-induced changes in the surrounding microenvironment (53). Similarly, multimodal profiling combines transcriptomic and morphological measurements of compound-induced cell states and could provide complementary information that may be missed by either modality alone (54).

In summary, perturbational transcriptomics is the mechanism-of-action assessment step within an AIDD workflow that helps determine which candidate molecules, tumor subtypes, resistance states, or combination partners warrant continued validation. Perturbation profiles can indicate whether a candidate drug changes the target pathway and reverses the disease-associated cell state before more costly functional, animal, or combination experiments are performed. This helps eliminate molecules that simply bind the target or have a plausible chemical structure but do not produce the expected cell response. However, these limitations mean that the reliability of AI perturbation-response models depend not only on how they are trained and tested, but also on the biological relevance of the data used to generate and evaluate them. Finally, predicted gene-expression similarity, signature reversal, and pathway-activity changes should not be taken as proof of mechanism but rather hypotheses requiring experimental confirmation.

## 5 Drug sensitivity, resistance, and combination prioritization

The efficacy of candidate drugs can be further evaluated with AI-driven drug response models. These models estimate the biological contexts in which meaningful therapeutic activity is most likely to emerge, how resistance may develop, and whether drug combination strategies may improve efficacy. Unlike perturbational transcriptomics, response-prediction approaches estimate how candidate therapeutics behave across different, and complex disease models. In practice, they are most useful for assessing the efficacy, the translational potential and possible pitfalls of candidate drugs, and serve as a basis for experimental validation.

Early AI-driven drug response models relied heavily on pharmacogenomic resources and combinatorial screening datasets such as the Genomics of Drug Sensitivity in Cancer (GDSC) and Profiling Relative Inhibition Simultaneously in Mixtures (PRISM), which profile drug sensitivity using cancer cell lines (55, 56). For example, DrugCell is a deep learning model that is trained on the GDSC and related datasets to produce biologically interpretable drug sensitivity by integrating tumor genotypes from cell lines, drug structure, and pathway hierarchy (57). However, although GDSC and PRISM remain foundational training resources, they do not fully capture patient response. Several strategies have since improved the clinical translatability of AI-based drug response models. One such strategy is transfer learning, which adapts models trained on high-throughput cell-line screens to patient-derived tumor cells and xenografts, thereby narrowing the preclinical-to-clinical gap (58). Prediction accuracy has also improved through models that integrate more complex input data. Examples include MOLI (59) and Super.FELT (60), two multi-omics models that combine mutation, copy-number, and gene-expression data and extend drug-response prediction from cancer cell lines to patient-derived xenografts.

Importantly, AI models trained on patient-derived organoid pharmacogenomic data have predicted *in vitro* drug efficacy that translates to patient outcomes (61, 62). Unlike models trained on experimental *in vitro* drug responses, ENLIGHT, a genetic-interaction-based ML model, predicts treatment response directly from clinical datasets. Using treatment-naïve tumor transcriptomes, it predicts responses across blinded clinical trial datasets (63), while its image-based extension, ENLIGHT-DeepPT, infers transcriptomic information from histopathology to make similar predictions (64). While these models are useful for evaluating the translational potential of drugs, patient response is often complicated by their physiology, treatment history, and the evolution of drug resistance. Indeed, intratumoral heterogeneity allows resistant subpopulations of cells to persist through treatment and therefore bulk transcriptomics (an average of all cells) can mask their drug response in these models. The AI model PERCEPTION addresses this by bringing single-cell resolution to predict both patient response and the emergence of resistance from intratumoral signals (65). Treating resistant or heterogeneous tumors often requires drug combinations, so methods that predict effective combinations are becoming increasingly important. TranSynergy, a deep learning model, integrates drug targets, gene dependencies, and interaction networks to prioritize synergistic drug pairs (66). Several platforms now combine AI-based prediction with patient-derived validation. For example, the quadratic phenotypic optimization platform (QPOP) ranks individualized combinations using ex vivo lymphoma biopsies in relapsed or refractory disease with prospective clinical validation (67, 68). Similarly, scTherapy and related acute myeloid leukemia models identify drug combinations that co-inhibit resistant clones while preserving non-malignant cells (69, 70). Organoid-based Bayesian optimization has also demonstrated that different dosing regimens can alter the efficacy of drug combinations (71).

Together, the above approaches extend AIDD into increasingly disease-relevant systems by prioritizing drug response, resistance, and combination strategies. However, the utility of these models in a clinical setting remains limited due to several translational barriers. For example, most models have been validated retrospectively, using pre-existing data, and show reduced performance when evaluated on independent or clinically representative datasets (72, 73). Conversely, the application of prospective validation on blinded datasets, as performed with ENLIGHT and QPOP, has shown improved predictive potential in clinical settings. In addition, limited sample sizes, heterogeneous data quality, and overlapping training and validation datasets can lead to overfitting and poor generalization across patient populations. The use of prospective clinical validation and improvements in data quality may enable more reliable prediction of patient-specific drug responses and facilitate future clinical integration.

## 6 Current limitations and future directions for AI-driven cancer drug discovery

The four stages of AIDD reviewed here should be considered as interdependent stages of a single design-test-refine workflow. Indeed, stage 1 identifies therapeutic targets worth pursuing, stage 2 proposes candidate molecules against these targets, stage 3 establishes whether a molecule produces the intended cellular state, and stage 4 estimates sensitivity and resistance to the hit molecules as well as synergy between hit molecules in more disease-relevant systems. Each stage passes its output to the next, and experimental evidence generated at any point can feed back to refining earlier decisions. A consideration that applies across all four stages is that AI generates predictions or recommendations rather than validated results and is only a starting point for experimental work and not an endpoint. **Table 1** sets out, for each stage, the AI output, the corresponding validation standard, and the dominant translational risk that remains when validation is absent. The underlying point is that without experimental confirmation, an AI ranking can appear convincing while contributing little practical value.

While the outputs for each AIDD stage differ, they have several common limitations. Firstly, random data splitting can place closely related compounds or cell lines in both the training and test sets, inflating model performance. More informative benchmarking should evaluate models on unseen compounds or cell lines and compare them with simple baselines (52). In addition, most published drug response models perform no better than simple baselines when all models are given the same tuning budget, kept free of train–test overlap, and scored on the same metrics (72, 73). Indeed, reported performance should be interpreted against a clearly stated data split, a simple baseline, and extrapolation control. Secondly, most models optimize a proxy. While docking scores, transcriptional similarity, and synergy indices are readily computed, they do not necessarily have a therapeutic benefit. A third limitation is that the complexity of cancer biology, including intratumoral heterogeneity, drug resistance, tumor microenvironment, and normal-tissue toxicity, remains underrepresented in all four stages. A final limitation concerns the lack of integration of the four stages, since molecular design, perturbation profiling, patient-derived testing, and clinical annotation are rarely performed within a single experimental system. This makes the design-test-refine workflow difficult to implement end-to-end.

The future of cancer AIDD is likely to move toward closed-loop systems in which a single agent links target identification through to response prediction and feeds each result back into the next decision. Within such a framework, AI does not replace experimental cancer biology, but rather enables experimental choices to be made faster, more interpretable, and better aligned with the validation that cancer drug development demands.

## 7 Conclusion

AI tools are emerging as practical accelerators of cancer drug development, helping researchers decide which therapeutic targets to validate, which molecules to design, which pathways to target, and which treatment to prioritize. Their most reliable near-term value lies in suggesting efficient therapeutic strategies across increasingly complex biological, chemical, and patient-derived data.
